# Experiences and life circumstances of unintentionally pregnant women affected by intimate partner violence—stress factors, resources, healthcare structures and needs: a scoping review protocol

**DOI:** 10.3389/fpubh.2024.1422918

**Published:** 2024-10-16

**Authors:** Kristina Winter, Jana Niemann, Dennis Jepsen, Petra J. Brzank

**Affiliations:** ^1^Institute for Social Medicine, Rehabilitation Sciences and Health Services Research, Nordhausen University of Applied Science, Nordhausen, Germany; ^2^Institute of Medical Sociology, Interdisciplinary Centre for Health Sciences (PZG), Medical Faculty, Martin Luther University Halle-Wittenberg, Halle, Germany

**Keywords:** abuse, women, reproductive health, maternity care, abortion, pregnancy termination, domestic violence

## Abstract

This scoping review outlines the current understanding, challenges, available resources, and healthcare needs of women affected by intimate partner violence (IPV) who experience unintended pregnancy (UP). UPs are defined as unwanted, unplanned, or mistimed pregnancies. The impact of UP is multifaceted and carries several additional risks, particularly for women who experience IPV. The experiences and living conditions, including (mental) burdens, resources, care structures, and the needs of unintentionally pregnant women who have experienced IPV, remain mostly unexplored. The review will include the following criteria: (i) reproductive-aged women who have experienced IPV and UP; (ii) publications that provide detailed accounts of the experiences, circumstances, and/or needs of women with UP who have experienced IPV. This study will utilize the JBI methodology for scoping reviews and follow the PRISMA Protocol for Scoping Reviews (PRISMA-ScR). A total of 2,325 papers and gray literature published from 2000 to the present were identified. 1,539 literature items were included in the title and abstract screening. Two researchers will independently choose studies, perform data extraction, and perform data synthesis. Quantitative data will be narratively summarized and qualitative data will be analyzed using thematic analysis. The findings will identify research gaps and provide insights into an important topic of reproductive healthcare and the (mental) health situation of a particularly vulnerable group. This will be useful in defining indications for researchers, professionals, and policymakers in public, mental, and reproductive health to conceptualize interdisciplinary and empirical healthcare support for affected women.

**Systematic review registration:**

Open Science Framework: https://doi.org/10.17605/OSF.IO/ZMVPE.

## 1 Introduction

Unintended pregnancy (UP) is a stressful life event in which various resources, stresses, and coping strategies influence handling and decision-making. Internationally it has become standard to distinguish between “unwanted” and “mistimed” pregnancy, the generic term “unintended” (1, 2). Additionally, the need for an experimental design (UPs carried to term as a control group for terminating pregnancies) has been emphasized (1–4). Many factors can lead to UP, including inadequate sexual education, lack of information and/or access to safe contraception, unprotected sex, contraceptive failure or manipulation, sexual violence, and coercive control (4–9). The co-occurrence of elevated stress and higher UP rates also applies to women who have experienced violence or are pregnant in violent relationships (10, 11). This may cause unintended pregnancies among women who experience IPV (WEIPV), creating a significant public health issue that affects their health and wellbeing (12).

The Istanbul Convention’s (Council of Europe Convention on Preventing and Combating Violence against Women and Domestic Violence, 2011) definition of domestic violence (usually used simultaneously with IPV) is widely accepted as an international standard. It includes “all acts of physical, sexual, psychological or economic violence that occur within the family or domestic unit or between former or current spouses or partners, whether or not the perpetrator shares or has shared the same residence with the victim.,” whereas various forms of IPV frequently co-occur and are associated with one another. IPV is the most common form of violence against women (13). In 2021, approximately 81,100 women and girls worldwide died as a result of violence, with more than half of these deaths occurring in the hands of their intimate partners or close family members (excluding the number of unreported femicides) (14). Moreover, it is estimated that one in three women worldwide has experienced either physical and/or sexual violence and up to 40% psychological IPV in their lifetime (14–15).

The impact of UP is multifaceted and carries several additional risks, particularly for women who experience intimate partner violence (WEIPV). These risks can affect

- The health behavior, e.g., substance or nicotine use as a coping mechanism addressing the experienced IPV, or a less frequent attendance at appointments regarding prenatal care
- The reproductive health, e.g., increased risks of preterm births, low birth weight, fetal injuries, infections with sexually transmitted diseases, small gestational age, the conduction of unsafe abortions, and maternal death,
- As well as other aspects of physical and mental health, such as increased risks of maternal depression, anxiety, post-traumatic stress disorder, a disturbed bonding to the newborn and direct health consequences for the child (16, 17).

To provide practical indications regarding the conception of interdisciplinary care and support options for unintentionally pregnant WEIPV, it is essential to put these findings regarding increased health risks into context. Firstly, contrary to the popular persistent and empirically unendorsed concept of “post-abortion syndrome,” international studies show that there is no lasting risk to women’s mental health from intentional first-trimester abortion (9, 18, 19). In contrast, mental health problems before pregnancy play an important role in decision-making and coping with pregnancy outcomes (termination/delivery) (20), which should be considered, especially within case conception. Secondly, social resources, or the ability to be embedded in a social network, can have a positive impact on coping with UPs and supporting decision-making. For example, this can be achieved through practical support, as well as the possibility of engaging in discourse and reflection on issues and problems with a close person, which in turn can also lead to stress reduction (21). At the same time, they can also have a negative impact (22, 23), for example, if pressure or coercion is exerted on the individual to decide on the carrying or abortion of the pregnancy. This is compounded by the confrontation with societal perceptions and legal frameworks (22–24) that, among other things, make it difficult to access information and decide how to deal with an UP and are often accompanied by stigma, shame, and feelings of guilt (23–25).

## 2 Objectives and research questions

The experiences and living conditions, such as burdens, resources, care structures, and needs (e.g., socioeconomic position, family situation, family/social support, stigma, experiences with medical practitioners and the counseling system, and IPV-related conditions) of unintentionally pregnant women (birth or abortion) who have experienced IPV, remain largely unexplored. The study “Experiences and Living Conditions of Unintentionally Pregnant Women—Counseling and Care Services” for vulnerable groups (ELSA-VG) aims to describe and identify resources and burdens of unintentionally pregnant women with IPV experiences and their support and care needs.^1^ A scoping review of this topic will provide a comprehensive overview of existing knowledge on the psychosocial and healthcare situation, burdens, resources, and effectiveness of counseling and support for and in dealing with unintended (carried to term/aborted) pregnancies and WEIPV, thus making an important contribution to the field of sexual and reproductive health for a particularly vulnerable group (women affected by IPV). A preliminary search of PROSPERO and MEDLINE (PubMed) databases did not reveal any recent reviews on this topic. To the best of our knowledge, this review is the first to evaluate literature on this topic. Therefore, this review aims to examine (inter-)relationships between (1) IPV (and individual forms) and UP, (2) decision-making regarding UP (birth or abortion), and (3) the life circumstances of affected women to identify the specific challenges faced by WEIPV. The following research questions were used:

RQ1: What experiences and/or life circumstances are associated with IPV and with the decision-making process regarding the UP of WEIPV?

RQ2: What needs for support can be derived from unintentionally pregnant women who have experienced IPV?

Therefore, this review summarizes the current state of research, shows knowledge gaps, and informs what needs for support can be derived from this, especially for policy, practice, and further research [e.g., potential modifications to the counseling/care structure for WEIPV, important further analyses to understand this vulnerable group to improve their sexual and reproductive health (care), as well as their mental wellbeing].

## 3 Methods and analysis

For this scoping review (protocol), we will follow the JBI Methodology for Scoping Reviews (26, 27), Preferred Reporting Items for Systematic Reviews and Meta-Analysis Protocols (28) (PRISMA-P), and PRISMA Protocol for Scoping Reviews (29) (PRIMSA-ScR).

### 3.1 Eligibility criteria

The PCC mnemonic is employed to shape our eligibility criteria, considering the Population, Concept, and Context:

#### 3.1.1 Population

We will include articles that report the experiences and/or life circumstances (sociodemographic, socioeconomic, and psychosocial factors) of reproductive-aged women who have experienced both intimate partner violence and UP.

#### 3.1.2 Concept

Our scoping review will include publications that describe the experiences and/or circumstances of unintentionally pregnant WEIPV. This includes problems, resources, healthcare structures, and needs experienced by WEIPV with UP. Studies must either describe the distribution/prevalence of experiences and/or circumstances (quantitative studies) or how they are expressed by unintentionally pregnant WEIPV (qualitative studies). All reported experiences and/or life circumstances of unintentionally pregnant WEIPV, their relationship with UP, and their need for support will be extracted.

#### 3.1.3 Context

We will include publications that report the experiences and/or life circumstances of WEIPV in unintended pregnancies and their need for support.

In addition, we limited our sources for inclusion to any study design that considered the experiences and/or circumstances of WEIPV with UP: peer-reviewed studies (empirical studies, literature reviews), commentaries, editorials, report frameworks, guidelines, letters, and conference abstracts. Grey literature was also included after a quality check using the ACCODS checklist (30).

### 3.2 Search strategy

We used the keywords, index, and MeSH terms identified in a preliminary exploratory search of MEDLINE (EBSCOhost) to inform our search strategy (Supplementary Table 1). This search strategy was then translated and adapted for use in the remaining databases and grey literature sources (Embase, PsychInfo (EBSCOhost), CINHAL (EBSCOhost), and Google Scholar). On the 9th of January 2024, we searched all databases from 2000 to the present. As the subject of violence against women and the area of sexual and reproductive health garnered increasing attention, and the first state action plans were developed around the turn of the millennium, the year 2000 was selected as the starting point for this research (31). Our search will not be limited by language because of the availability of translation services (e.g., DeepL). Finally, we will manually screen all reference lists of the included studies to identify further studies that met our inclusion criteria.

### 3.3 Study selection

**Figure 1:**
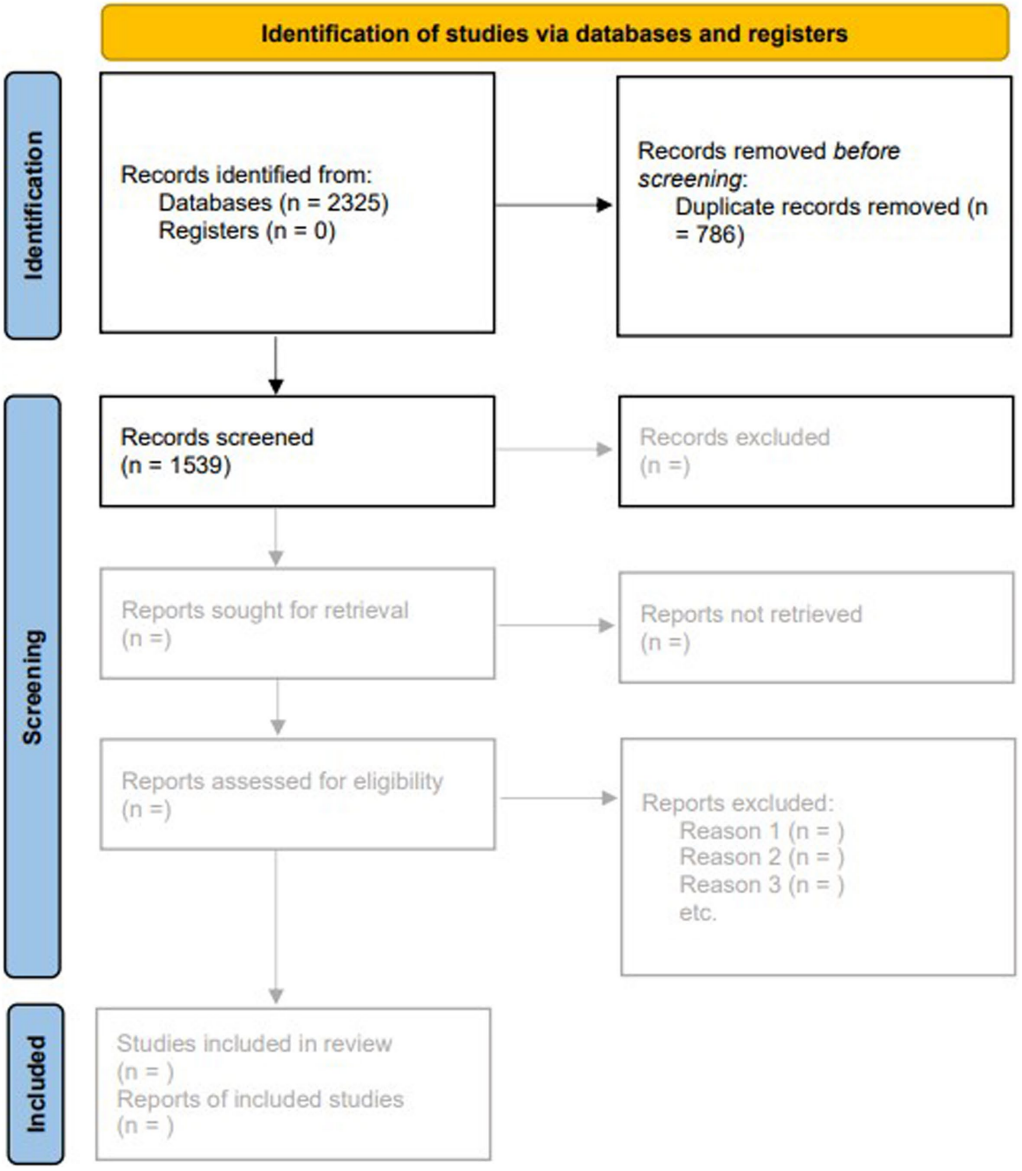
PRISMA flow chart. PRISMA, Preferred Reporting Items for Systematic Reviews and Meta-Analyses.

After the search, all publications (*N* = 2,325) were transferred to Rayyan (32) (Qatar Computing Research Institute, Doha, Qatar), and duplicates (*n* = 786) were removed. 1,539 literature items were included in the title and abstract screening (*cf.* Figure 1). To ensure rigor and consistency, two researchers (KW and JN) will independently screen all articles for titles, abstracts, and full texts. Disagreements between reviewers during this process will be discussed by the research team until consensus is reached. The reasons for exclusion of studies during full-text screening will be documented. The study selection process will be documented and summarized in a PRISMA flow diagram.

### 3.4 Data extraction and coding

We will import all included studies into MAXQDA to code the documents for data extraction. The codes will be based on a data extraction table. The table will include information, such as authors, study design, geographic location, study objective, participant information (e.g., pregnancy status at the time of study), study setting, reported experiences, and circumstances of WEIPV with unintended pregnancies. The data extraction table will be refined during the extraction process. Any changes will be documented in the final scoping review. Qualitative data will be coded inductively in an iterative process between KW and JN. The included studies will be equally divided between the two researchers (KW and JN). KW and JN will independently code a random sample of 10% of the included articles as a pilot to ensure rigorous and consistent data extraction. Any disagreements between KW and JN will be resolved through a discussion. If consensus cannot be reached, a final decision will be made within the research team.

### 3.5 Data analysis

We will synthesize the general characteristics of the included studies (e.g., geographic location, participants, and study setting) using frequencies, percentages, and narrative descriptions. The prevalence of reported experiences and/or circumstances of unintentionally pregnant WEIPV will be reported using descriptive statistics for quantitative outcomes. Thematic analysis will be performed to identify the means and patterns among the qualitative studies.

## 4 Discussion

UP is a complex and widespread issue globally and poses multiple risks to women, which can have a significant impact on women’s (mental) health. Therefore, this is an important public (mental) health concern. Women who have experienced partner violence are a particularly vulnerable group in this regard; for example, IPV can increase the likelihood of UP and, at the same time, an (unintended) pregnancy can increase the risk of IPV. This scoping review aims to examine the evidence surrounding the understanding, challenges, available resources, and healthcare needs of unintentionally pregnant women who experience IPV. The results of this review will synthesize the key findings of the available studies to present the current state of research on unintentionally pregnant women with experiences and circumstances of intimate partner violence, and to identify the support needs that can be derived. We will offer a comprehensive overview of peer-reviewed publications, including quantitative and qualitative studies. To provide an interdisciplinary support system for WEIPV with UP addressing their reproductive, mental, and physical health it is to identify responsibilities and challenges for researchers, professionals, and policymakers based on this review’s findings. Additionally, it is essential to recognize areas where collaboration between these groups is necessary.

## 5 Strengths and limitations

To the best of our knowledge, this is the first scoping review that addresses healthcare needs and conceptual challenges for interdisciplinary care of unintentionally pregnant women affected by IPV. This review will provide an overview of the individual life circumstances and health of affected women and set them into context with the requirements of the Istanbul Convention, as well as the Group of Experts on Action against Women and Domestic Violence (GREVIO).

A potential limitation of the scoping review is the absence of an assessment of the methodological quality of the included studies. Nevertheless, a scoping review is the preferred methodology for gaining a comprehensive understanding of the existing literature on the research topic and the current state of knowledge in the field. Although a methodical approach will be employed to examine grey literature, the sheer volume of available material means that some sources may have been overlooked; we do understand this as a limitation. Additionally, although we will use readily available translators, they might not be available in all languages.

## 6 Ethics and dissemination

No ethical approval is needed for the scoping review. The results of this scoping review will be summarized and published in a peer-reviewed journal. The final report will follow the PRISMA-ScR guidelines and visually present the synthesized evidence through tables, diagrams, and direct quotes from qualitative studies.

## Footnotes

^1^ https://www.elsa-nordhausen.de/
