# Supplementary material for: Experiences and life circumstances of unintentionally pregnant women affected by intimate partner violence—stress factors, resources, healthcare structures and needs: a scoping review protocol
Source: Front Public Health. 2024 Oct 16;12:1422918. doi: 10.3389/fpubh.2024.1422918 (PMC11521957; doi:10.3389/fpubh.2024.1422918)
Supplement: Supplementary file 1 [file Table_1.DOCX]

**Appendix 1: Search strategy conducted in PubMed January 09^th^, 2024**

| **Search** | **Query** | **Records retrieved** |
| --- | --- | --- |
| **Medline (PubMed)** | | |
| #1 | ("unintended"[Title/Abstract] OR "unwanted"[Title/Abstract] OR "unplanned"[Title/Abstract] OR "terminated"[Title/Abstract]) AND (fha[Filter]) AND (2000:2024[pdat]) | 81,163 |
| #2 | (partner violence[Title/Abstract] OR intimate partner violence[Title/Abstract] OR partner abuse[Title/Abstract] OR violence during pregnancy[Title/Abstract] OR domestic violence[Title/Abstract] OR abused women[Title/Abstract] OR battered women[Title/Abstract] OR reproductive coercion[Title/Abstract] OR birth control sabotage[Title/Abstract] OR contraception sabotage[Title/Abstract] AND (fha[Filter]) AND (2000:2024[pdat])) | 18,102 |
| #3 | (pregnan*[Title/Abstract] OR abort*[Title/Abstract] OR pregnancy termination[Title/Abstract] OR voluntary pregnancy interruption[Title/Abstract] AND (fha[Filter]) AND (2000:2024[pdat])) | 412,318 |
| #4 | (Pregnancy OR Abortion, Induced[MeSH Terms] AND (fha[Filter]) AND (2000:2024[pdat])) | 540,393 |
| #5 | (Intimate Partner Violence OR Battered Women OR Domestic Violence[MeSH Terms] AND (fha[Filter]) AND (2000:2024[pdat])) | 38,527 |
| #6 | (Conflict, Psychological OR Family Conflict OR Stress, Psychological AND (fha[Filter]) AND (2000:2024[pdat])) | 291,896 |
| #7 | #1 AND #2 AND #3 | 349 |
| #8 | #4 AND #5 AND #6 | 431 |
| #9 | #1 AND #2 AND #3 AND #4 AND #5 AND #6 | 20 |
